# Age at menarche in relation to nutritional status and critical life events among rural and urban secondary school girls in post-conflict Northern Uganda

**DOI:** 10.1186/1472-6874-14-66

**Published:** 2014-05-09

**Authors:** Beatrice Odongkara Mpora, Thereza Piloya, Sylvia Awor, Thomas Ngwiri, Paul Laigong, Edison A Mworozi, Ze’ev Hochberg

**Affiliations:** 1 Department of Paediatrics & Child Health, Paediatric Endocrinologist, Paediatrician, Lecturer, Gulu University, Faculty of Medicine, Gulu, Uganda; 2 Department of Paediatrics, Paediatric Endocrinologist, Paediatrician, Lecturer, Makerere University College of Health Sciences, School of Medicine (MakCHS), Kampala, Uganda; 3 Department of Reproductive Health, Obstetrician & Gynaecologist, Lecturer, Gulu University Faculty of Medicine, Gulu, Uganda; 4 Paediatric Endocrinologist, Pediatrician, PETCA Gertrude’s Children’s Hospital, Nairobi, Kenya; 5 Senior Consultant Paediatrician, Mulago National Referral Hospital, Honorary Lecturer MakCHS, Kampala, Uganda; 6 Professor Emeritus of Paediatrics and Endocrinology, Technion, Israel Institute of Technology, Haifa, Israel

**Keywords:** Menarche age, Nutritional status, Body composition, Stress, Uganda

## Abstract

**Background:**

Menarche age is an important indicator of reproductive health of a woman or a community. In industrial societies, age at menarche has been declining over the last 150 years with a secular trend, and similar trends have been reported in some developing countries. Menarche age is affected by genetic and environmental cues, including nutrition. The study was designed to determine the age at menarche and its relation to childhood critical life events and nutritional status in post-conflict northern Uganda.

**Methods:**

This was a comparative cross-sectional study of rural and urban secondary school girls in northern Uganda. Structured questionnaires were administered to 274 secondary school girls, aged 12 – 18 years to determine the age at menarche in relation to home location, nutritional status, body composition and critical life events.

**Results:**

The mean age at menarche was 13.6 ± 1.3 for rural and 13.3 ± 1.4 years for urban dwelling girls (t = -1.996, p = 0.047). Among the body composition measures, hip circumference was negatively correlated with the age at menarche (r = -0.109, p = 0.036), whereas height, BMI and waist circumference did not correlate with menarche. Paternal (but not maternal) education was associated with earlier menarche (F = 2.959, p = 0.033). Childhood critical life events were not associated with age at menarche.

**Conclusions:**

Age at menarche differed among urban and rural dwelling school girls and dependent on current nutritional status, as manifested by the hip circumference. It was not associated with extreme stressful childhood critical life events.

## Background

Over the past 150 years, the age of menarche has fallen by a full four years in the industrialized West. Much as the secular trend in human size has been an adaptive response to a nutritionally rich environment, the receding age of adolescence and pubertal development has been an adaptive response to positive environmental cues in terms of energy balance [1]. The current mean age at menarche in the United States of America is at 12.55 and 12.0 years among black and white girls, respectively [2]. In Sub-Sahara Africa, a secular trend in age at menarche has also been observed in several countries, with socioeconomic status, altitude, ethnicity and food security playing an important role. A study in Ethiopia compared girls with food security and those without and found a significant earlier age at menarche among the former [3]. Similar results were reported from the Amazonia, Senegal and India [4-6].

When children experience severe environmental stresses such as malnutrition or disease, maturation is delayed until conditions improve and normal growth can resume, which may be regarded as a wait-and-see strategy for environmental conditions to improve before the affected individual assumes an energetic costly gestation. Hochberg and Belsky have recently stipulated that familial psychosocial stress (e.g., marital conflict, harsh parenting, father absence) or extra-familial ecological stress (e.g., limited income, unemployment) fosters a fast rather than quality-oriented reproductive strategy; psychosocial stress accelerates pubertal maturation and, relatedly, sexual debut, promiscuity and parental investment [7].

Here we tested the puberty-related theory of socialization, in post-conflict northern Uganda in adolescent girls who had been subjected to extreme hardship of war and poverty. Gulu District had been at the center of a two-decade rebellion between the Lord’s Resistance Army led by Joseph Kony and the army of the government of Uganda. More than 95% of the population was displaced into camps with no access to their farmland. Malnutrition was common and affected mostly children and pregnant mothers. Many children were abducted and used as child soldiers or sex slaves by the rebels [8]. Many lost their relatives and were forced to kill others, hence suffered from extreme psychological stress [9]. Girls in rural areas were more at risk of stressful critical life events than those in urban areas; they were more susceptible to abduction, displacement, poverty and malnutrition.

The working hypotheses assumed that (i) girls stressed during their childhood will have earlier menarche; (ii) current nutritional status will correlate with menarche age; (iii) girls in rural environment will have earlier age at menarche than those in urban homes.

## Methods

### Setting

Gulu District, located 332 km north of the capital Kampala, has an area of 12,000 km^2^ and a population of 354,000, with a male/female ratio of 0.96. The main economic activity is farming, and poverty is widespread and severe; most families survive on either external aid or less than a dollar a day. The region was involved in a war for over 20 years from 1985 to 2006. The study subjects were born and raised during the war and were between 6 and12 years of age at the end of the war. The study subjects were selected from 11 rural and 11 urban secondary schools, which were randomly sampled. Rural girls live in villages without access to electricity, running water or roads, while urban girls live in areas with electricity, water, roads and healthcare facilities within a 5-kilometer radius from town centers.

### Subjects

The design criteria included all the girls in the selected schools, except those suffering from chronic illnesses. A systematic sampling method was employed to ensure equal chance to participate in the study. Sample size was calculated comparing two means, assuming the power of 80% to detect any significant difference between the two groups [10], and required a total of 274 subjects. A total of 274 students, aged 12 – 18 years were studied with 137 subjects in the rural and 137 in the urban group. Three girls were excluded because they had not had their menarche and 271 were subjected to the final analysis. A set of standardized pre-coded questionnaires were used to obtain menarche data and demographic characteristics. The questionnaires were self-administered to the girls while at school.

The dependent variable was age at menarche and independent variables included demographic characteristics: place of residence, family size, the presence of siblings and parents in the household, maternal and paternal education, maternal and paternal occupation, parents’ marital status, BMI, as well as a list of stressful life events that occurred during the war. Such stressful events include abduction into rebel captivity, loss of close first-degree relatives during the war, living in displacement camps and not living with both parents in the same household.

Place of residence was defined as where the girls lived with the rest of the family members at the time of the interview. Family size was defined as the number of persons in the family, while paternal and maternal education implied the level of academic achievements of the parents specified as no education, primary, secondary and tertiary education. Parental occupation was defined according to the job or work each parent did to earn a living and take care of the family and it was specified as peasant farmer, employed, housewife or unemployed. The parents were considered married if they were in a matrimonial relationship and living together in the same household.

### Statistics

Data entry was done using EPI Data 3.1 and analysis was done by SPSS version 17.1. A bivariate analysis was done to compare the characteristics of secondary school girls in urban and rural schools with respective p values for chi-square. An independent sample t-test was done for two independent groups to compare age at menarche among rural and urban school girls in relation to various critical life events. Nutritional status for rural and urban school girls was compared using the independent samples t-test. One-way ANOVA was used to compare menarche age with parental parameters. Each stressful life event was analyzed independently for association with age at menarche. Pearson’s correlation analysis was conducted on the combined analysis for the relationship between age at menarche and independent variables as listed above. Statistical significance was defined as p-value <0.05.

### Ethical consideration

Ethical approval was obtained from the Ethics Committee of Gulu University Faculty of Medicine. Parents were informed in writing about the study and agreed verbally for their daughters to be interviewed. Informed consent was obtained from the school administration and verbal assent was obtained from participating students. Girls with menarche problems and other pubertal problems were further investigated and treated by an endocrinologist.

## Results

### Characteristics of study participants

Table 1 shows the 271 high school girls, divided into rural and urban. The majority of the girls, 143/271(52.8%), had mothers with primary school education: 65% and 40.3% being rural or urban, respectively. Girls in urban schools had more mothers with secondary and tertiary education (36.6% and 19.7%, resp.) than their rural counterparts (11.9 and 2.2%, resp. (*X*^*2*^ *= 24.072,* p < 0.001). The majority of the fathers of urban and rural school girls had secondary or tertiary education (42.5% *vs*. 48.9% and 35.1% *vs.* 9.5%, resp. *X*^*2*^ *= 41.520*, p < 0.001). The majority of the girls in both rural and urban schools had elder siblings of both sexes in the same households. Normal BMI was present in 80.3% and 82.3% for rural and urban girls, resp. (NS). The most reported stressful critical life events were: living in ‘internally displaced persons’ (IDP) camps (56%), loss of relatives during the war (77.1%), and not living with both parents in the same households (54.2%). There were more abducted girls in rural areas (*X*^*2*^ *= 4.404,* p = 0.038).

**Table 1:** Demographic characteristics of study participants by school location

**Characteristics**		**N (%)**	**Urban**	**Rural**	**p-value**
			**N (%)**	**N (%)**	
School location		271 (100)	134 (50)	137 (50)	0.065
Home Location		271 (100)	117 (43.2)	154 (56.8)	<0.001
Age at Menarche: Mean ± s.d (yrs)		13.48 ± 1.48	13.36 ± 1.45	13.61 ± 1.27	0.139
Lives with both parents	No	147 (54.2)	73 (54.5)	74 (54.0)	<0.001
	Yes	124 (47.8)	61 (45.5)	63 (46.0)	
Has brothers	No	20 (7.4)	11 (8.2)	9 (6.6)	0.388
	Yes	251 (92.6)	123 (91.8)	128 (93.4)	
Has Elder brother:	No	93 (37.1)	45 (36.6)	48 (37.5)	0.492
	Yes	158 (62.9)	78 (63.4)	80 (62.5)	
Has sisters	No	21 (7.7)	8 (6.0)	13 (9.5)	0.196
	Yes	250 (92.3)	126 (94.0)	124 (90.5)	
Has elder sisters	No	102 (48.1)	45 (36.6)	57 (46.3)	0.183
	Yes	114 (51.9)	78 (63.4)	66 (53.7)	
Lived in IDP camps	No	120 (44.3)	80 (59.7)	40 (29.2)	<0.001
	Yes	151 (55.7)	54 (40.3)	97 (70.8)	
Ever been abducted by LRA	No	242 (89.3)	125 (93.3)	117 (85.4)	0.025
	Yes	29 (10.7)	9 (6.7)	20 (14.6)	
Lost Relative (s) during the war	No	62 (22.9)	41 (30.6)	21 (15.3)	0.002
	Yes	209 (77.1)	93 (69.4)	116 (84.7)	
Nutritional status:					
	Underweight	16 (5.9)	8 (6.1)	8 (5.8)	0.470
	Normal	220 (81.2)	106 (80.3)	114 (82.0)	
	Overweight	33 (12.2)	18 (13.6)	15 (10.8)	
	Obese	2 (0.7)	0 (0.00)	2 (1.4)	
Maternal education					
	No education	33 (12.2)	15 (11.2)	18 (13.1)	<0.001
	Primary	143 (52.8)	54 (40.3)	89 (65.0)	
	secondary	76 (28.0)	49 (36.6)	27 (19.7)	
	Teriary	19 (7.0)	16 (11.9)	3 (2.2)	
Maternal Occupation					
	Peasant farmer	195 (72.0)	79 (59.0)	116 (84.7)	<0.001
	Employed	35 (12.9)	29 (21.6)	6 (4.4)	
	Self Employed	41 (15.1)	26 (19.4)	15 (10.9)	
Paternal education					
	No education	20 (7.4)	14 (10.5)	6 (4.4)	<0.001
	Primary	67 (24.7)	16 (11.9)	51 (37.2)	
	secondary	124 (45.8)	57 (42.5)	67 (48.9)	
	Teriary	60 (22.1)	47 (35.1)	13 (9.5)	
Paternal Occupation					
	Peasant farmer	133 (49.1)	52 (38.8)	81 (59.1)	0.004
	Employed	92 (33.9)	55 (41.0)	37 (27.0)	
	Self Employed	46 (16.9)	27 (20.2)	19 (13.9)	

### Age at menarche

The mean age at menarche was 13.6 ± 1.3 for rural and 13.3 ± 1.4 years for urban dwelling girls (t = -1.996, p = 0.047).

### Critical life events and age at menarche

As evident from Table 2, critical life events were not associated with age at menarche. Paternal (but not maternal) education was associated with earlier menarche (F = 2.959, p = 0.033).

**Table 2:** The effects of war and critical life events on age at menarche among urban and rural school girls

**Factors**		**Sample (N)**	**Age at menarche (Years ± SD)**	**p-value**
Home Location	Urban	117	13.29 ± 1.43	0.047*
	Rural	154	13.63 ± 1.29	
School location	Urban	137	13.36 ± 1.45	0.139
	Rural	134	13.61 ± 1.27	
Lives with Both Parents	No	147	13.40 ± 1.28	0.280
	Yes	124	13.58 ± 1.45	
Presence of brothers	No	20	13.50 ± 1.57	0.966
	Yes	251	13.48 ± 1.36	
Presence of elder brothers	No	93	13.58 ± 1.44	0.369
	Yes	158	13.42 ± 1.35	
Presence of Sister	No	21	13.86 ± 1.39	0.329
	Yes	150	13.46 ± 1.36	
Presence of elder Sister	No	102	13.46 ± 1.35	0.997
	Yes	144	13.46 ± 1.38	
Lived in camps	No	120	13.53 ± 1.49	0.647
	Yes	151	13.45 ± 1.26	
Abducted by LRA rebels	No	242	13.45 ± 1.40	0.211
	Yes	29	13.78 ± 0.96	
Loss of relative(s) during war	No	62	13.22 ± 1.34	0.077
	Yes	209	13.56 ± 1.36	
Maternal Education	No education	33	13.52 ± 1.49	0.705
	Secondary	76	13.49 ± 1.25	
	Tertiary	19	13.82 ± 1.05	
Paternal Education	No education	20	13.65 ± 1.15	0.033*
	Primary	67	13.19 ± 1.38	
	Secondary	124	13.43 ± 1.34	

* = P < 0.05 was considered statistically significant.

### Nutritional status and age at menarche

**Figure 1:**
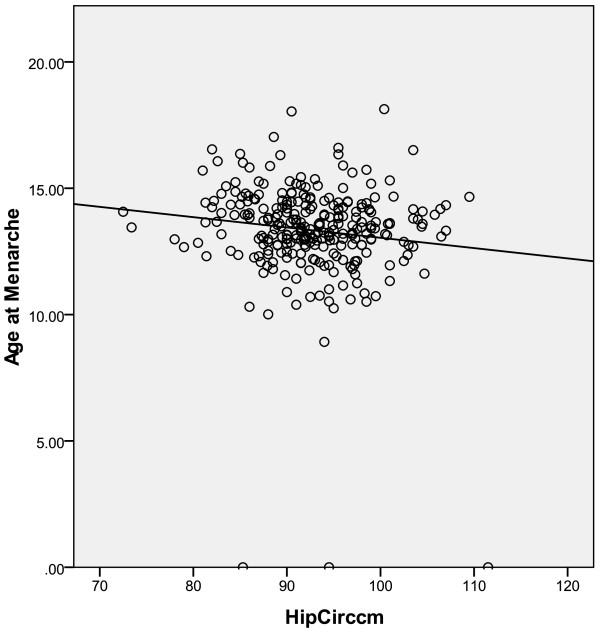
Age at menarche as a function of hip circumference.

Generally, the anthropometric measurements and nutritional indices were lower among rural secondary school girls than those of their urban counterparts, but height, BMI and waist circumference did not become statistically significant. There was a significant difference in hip circumference among rural and urban secondary school girls (t = 2.082, p = 0.038) and the hip circumference was negatively correlated with menarche age (r = -0.109, p = 0.036, Tables 3 and 4, Figure 1).

**Table 3:** Age at menarche and current nutritional status of rural & urban secondary schools (mean ± SD)

**Characteristics**	**Rural**	**Urban**	**p-value**
**Age at menarche**	13.63 ± 1.29	13.29 ± 1.43	0.047*
**Home location**	13.61 ± 1.27	13.36 ± 1.45	0.139
**School location**			
**Weight (Kg)**	56.24 ± 6.89	57.63 ± 7.55	0.112
**Height (cm)**	162.00 ± 5.89	162.78 ± 5.64	0.266
**Height (m2)**	2.63 ± 0.19	2.65 ± 0.18	0.247
**BMI (kg/m2)**	21.35 ± 2.08	21.74 ± 2.57	0.169
**Waist Circ (cm)**	72.28 ± 5.04	73.03 ± 6.18	0.272
**Hip circ (cm)**	91.95 ± 5.57	93.49 ± 6.60	0.038*
**Waist/Hip ratio**	0.79 ± 0.06	0.78 ± 0.06	0.697

*= p-value <0.05 was considered statistically significant.

**Table 4:** The pearson correlation between age at menarche and body composition

**Anthropometric**	**Pearson**	**p-value**
**measures**	**correlation (r)**	
Weight	-0.056	0.179
Height	+0.032	0.301
BMI	-0.083	0.087
Waist circumference (cm)	-0.017	0.391
Hip circumference (cm)	-0.109	0.036*
Waist/Hip Ratio	+0.078	0.100

* = p-value <0.05 is statistically significant.

## Discussion

Previous studies, as recently reviewed [2], have shown that the nutritional status and the social context of an adolescent girl, such as the family composition, have important influence on the menarche age. It was also shown that the absence of a biological father, the presence of half-brothers and step-brothers, and living in an urban environment are associated with earlier menarche, while menarche age was not affected by the number of brothers in the household, nor was there an effect of birth order [11]. Whereas our study, which was carried out in the unique situation of an African provincial post-war region, confirms the importance of body composition and confirms the earlier menarche of urban girls, we find no impact of an extreme stressful childhood life events on menarche age.

Why is it that adolescent girls of northern Uganda showed such resilience to extreme hardship during their childhood, abduction by rebels and loss of relatives to the war, which did not affect age at menarche? This may be due to a selection bias to the study of girls who attend secondary schools, indicating a higher social class; more than half of the girls in this region drop out of school before reaching secondary school levels due to extreme poverty that forces them to early marriage and child labor.

Indeed, within this group paternal (but not maternal) education was associated with earlier menarche. To understand the findings, we resort to Del Giudice’s integrated evolutionary model of the development of attachment and reproductive strategies [12]. His argument called for two critical ages during late infancy and middle childhood that have adaptive significance for reproductive strategies, including menarche age. Late infantile psychosocial stress and insecure attachment switch development towards early puberty and early mating-oriented reproductive strategies. During middle childhood insecure males adopt avoidant strategies, i.e., they avoid engaging in early sexual activities, whereas insecure females tend to adopt anxious/ambivalent strategies, i.e., early menarche and initiation of sexual activities and reproduction to ensure survival, shifting to dismissing patterns when environmental risk is more severe and/or social resources are scarce. Paternal education is therefore associated with improved household income and since fathers are the greatest contributors and determinants of household income, the lack of paternal education is associated with unemployment, marital conflict and poverty which in turn are stressful life events affecting menarche age [7].

In our study, we also found that urban girls had earlier menarche, in support of previous reports from other countries. Furthermore, children were displaced from the periphery of towns into the town centers as night commuters with minimal adult supervision and hence early initiation of sexual activities, while girls in rural areas lived in IDP camps where they lived with parents [8,9].

Of the body composition variables, hip circumference stands out with a negative correlation with the age at menarche and significantly discriminating urban girls from rural girls in terms of menarche age. Hip circumference is a unique parameter in body composition, normally greater in females than in males; although directly correlated with BMI, it seems to support a specific protective metabolic role. The gluteo-femoral body fat has been shown to be inversely associated with blood glucose, blood pressure and lipids, and to be associated with reduced risk of CVD, all-cause and cardiovascular mortality [13]. This population-based survey among south Asian and African Mauritians showed that the well-documented waist circumference becomes strongly correlated with mortality only after adjustment for hip circumference and vice versa. More mechanistically, a beneficial adipokine profile is associated with gluteo-femoral fat; leptin and adiponectin levels were positively associated with gluteo-femoral fat while the levels of inflammatory cytokines were negatively associated [14]. It was previously suggested that larger hip circumference reflects greater muscle mass [15]. Manolopolous et al. suggested that “gluteo-femoral fat may protect our bodies, irrespective of gender, by trapping excess fatty acids and preventing chronic exposure to elevated lipid levels” [15]. Interestingly, among hunter-gatherers from north Tanzania, the Hadza men preferred women with “protruding buttocks” [16]. The results presented here support a previous contention that gluteo-femoral fat may be more relevant to menarche age than total fat [17]. Among American girls, an increase in gluteo-femoral fat was associated with an increased likelihood of being at menarche compared to fat distribution in other parts of the body [18]. Cross-sectional data from the third National Health and Nutrition Examination Survey (NHANES III) for females aged 10–14 showed that unit increases in hip circumference were associated with 24% higher odds of menarche, while increases in waist circumference and triceps skinfold lower the odds by 7 and 9%, respectively. On the other end of reproductive life, change from pre- to postmenopausal status is associated with increased waist circumference but reduced hip circumference [19].

In Uganda, little has been done in the area of menarche age, and the national age at menarche is not known. This is the first report of menarche age, and it confirms the urban–rural difference, as reported elsewhere.

The study has several inert limitations: Stressful life events might have been underreported because of fear, past experiences and stigma, especially abduction into rebel captivity. In addition, the degree of stress of such life events was not measured. Finally, girls in the same age group who have dropped out of school were not captured in this study.

## Conclusions

Hereditary, environmental and stochastic factors regulate puberty in a unique environment, but their relative contribution to the age at menarche is not known. We emphasize uniqueness of each child in her given genetic background and current environment as they best serve her reproductive fitness. The resilience of pubertal development among Ugandan girls who have been subjected to stressful life events would be better understand by studies of socio-nutritional factors as they interact with or via endocrine mechanisms in generating signals that affect menarche age.

## Competing interests

The authors declare that they have no competing interests.

## Authors’ contributions

OBM: Study design, data collection, analysis and report writing. TP: study design, data analysis. SA: Data Entry and analysis. TN: Study Supervisor, design and analysis. PL: Study co-supervisor, design and analysis. EAM: Study Supervisor, design and analysis. ZH: Study Supervisor, design, analysis, report writing. All authors read and approved the final manuscript.

## Pre-publication history

The pre-publication history for this paper can be accessed here:

http://www.biomedcentral.com/1472-6874/14/66/prepub
